# A SARS-CoV-2 cytopathicity dataset generated by high-content screening of a large drug repurposing collection

**DOI:** 10.1038/s41597-021-00848-4

**Published:** 2021-02-26

**Authors:** Bernhard Ellinger, Denisa Bojkova, Andrea Zaliani, Jindrich Cinatl, Carsten Claussen, Sandra Westhaus, Oliver Keminer, Jeanette Reinshagen, Maria Kuzikov, Markus Wolf, Gerd Geisslinger, Philip Gribbon, Sandra Ciesek

**Affiliations:** 1 Fraunhofer Institute for Translational Medicine and Pharmacology ITMP, Hamburg, 22525 Germany; 2 University Hospital Frankfurt, 60590 Frankfurt am Main, Germany; 3 Pharmazentrum Frankfurt/ZAFES, Institut für Klinische Pharmakologie, Klinikum der Goethe-Universität Frankfurt, 60590 Frankfurt am Main, Germany; 4 Fraunhofer Institute for Translational Medicine and Pharmacology ITMP, 60596 Frankfurt am Main, Germany; 5 Fraunhofer Cluster of Excellence for Immune Mediated Diseases CIMD, Frankfurt am Main, 60596 Germany; 6 DZIF, German Centre for Infection Research, External partner site, 60596 Frankfurt am Main, Germany

**Keywords:** Drug screening, Viral infection, Small molecules

## Abstract

SARS-CoV-2 is a novel coronavirus responsible for the COVID-19 pandemic, in which acute respiratory infections are associated with high socio-economic burden. We applied high-content screening to a well-defined collection of 5632 compounds including 3488 that have undergone previous clinical investigations across 600 indications. The compounds were screened by microscopy for their ability to inhibit SARS-CoV-2 cytopathicity in the human epithelial colorectal adenocarcinoma cell line, Caco-2. The primary screen identified 258 hits that inhibited cytopathicity by more than 75%, most of which were not previously known to be active against SARS-CoV-2 *in vitro*. These compounds were tested in an eight-point dose response screen using the same image-based cytopathicity readout. For the 67 most active molecules, cytotoxicity data were generated to confirm activity against SARS-CoV-2. We verified the ability of known inhibitors camostat, nafamostat, lopinavir, mefloquine, papaverine and cetylpyridinium to reduce the cytopathic effects of SARS-CoV-2, providing confidence in the validity of the assay. The high-content screening data are suitable for reanalysis across numerous drug classes and indications and may yield additional insights into SARS-CoV-2 mechanisms and potential therapeutic strategies.

## Abstract (summary)

**Table:** 

Measurement(s)	cytotoxicity • killing by virus of host cell
Technology Type(s)	bright-field microscopy
Factor Type(s)	concentration • incubation time
Sample Characteristic - Organism	Homo sapiens

Machine-accessible metadata file describing the reported data: 10.6084/m9.figshare.13562570

## Background & Summary

*Coronaviridae* is a family of encapsulated single-stranded positive-sense RNA viruses that typically cause mild respiratory diseases such as the common cold in humans^1^. However, several β coronavirus strains have emerged over the last two decades that cause acute respiratory infections associated with high mortality, particularly in individuals with underlying health conditions. Three major outbreaks have occurred, the first caused by Severe acute respiratory syndrome coronavirus (SARS-CoV) in 2002/2003^2^, the second caused by Middle East respiratory syndrome coronavirus (MERS-CoV) in 2012^3^, and the latest caused by Severe acute respiratory syndrome coronavirus 2 (SARS-CoV-2), which was first recorded in December 2019 and was declared pandemic in March 2020^4^. At the time of writing (9^th^ December 2020), more than 69 million confirmed SARS-CoV-2 infections have been reported worldwide and more than 1.6 million people have died from the associated disease, COVID-19^5^.

As a complement to the safe and effective vaccines against SARS-CoV-2, the repurposing of existing drugs represents a pragmatic strategy for the treatment of COVID-19 patients^6^. Drug repurposing (drug repositioning) is advantageous in the face of rapidly-spreading emerging diseases because the pathway to approval is facilitated by pre-existing preclinical (and often clinical or post-marketing) data, existing production capacity and supply chains can be used, and new mechanisms of action can be discovered that may suggest potential drug combinations for enhanced efficacy by exploiting synergistic effects^7^. Furthermore, bioinformatics analysis has identified 66 druggable proteins based on the SARS-CoV-2/human host cell interactome, which will allow the selection of compounds that interfere with interactions essential in the viral replication cycle^8^. Regardless of the origin of drugs against SARS-CoV-2, properly controlled clinical safety and efficacy studies are still needed before the approval of pharmacological treatments for COVID-19.

Multiple interventional clinical trials have been initiated in the search for effective treatments against SARS-CoV-2^9^. The individual drugs or combination treatments for these studies have often been selected based on known activities against SARS-CoV, Ebola virus, HIV or *Plasmodium* spp., hence the drugs under investigation include remdesivir, interferon β and ribavirin and lopinavir/ritonavir^10^. However, the search for effective drugs against SARS-CoV-2 could extend beyond known antivirals and anti-infectives if suitable high-throughput assays are used to identify candidates.

**Fig. 1:**
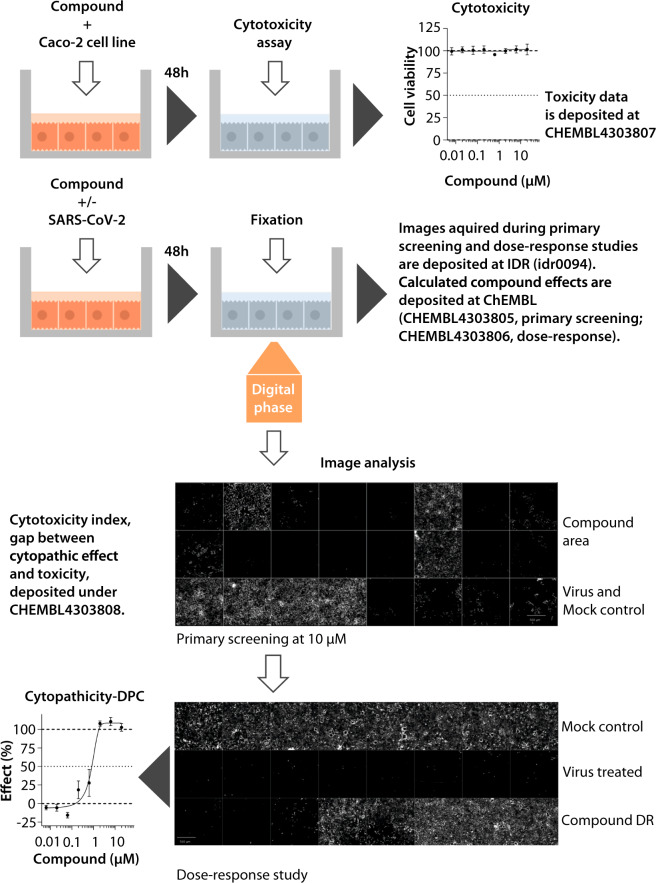
Cytopathicity assay workflow. To test for compound cytotoxicity, Caco-2 cells were grown to confluence and incubated with test compounds for 48 h. To test whether the compounds influenced the cytopathic effect of SARS-CoV-2, Caco-2 cells were grown to confluence and incubated with the test compounds and SARS-CoV-2 for 48 h. For image acquisition, the cells were fixed to comply with BSL1 requirements and imaged by digital phase contrast (DPC) microscopy. Upper image set shows the primary screen to determine cytotoxicity and cytopathic effect. Top two rows are representative wells of the compound area including three active molecules. Bottom row shows four positive control wells (virus treated, no compounds) and four negative control wells (no virus, no compounds). Lower image set shows the dose-response screen used to measure the anti-cytopathic effects of each compound at eight concentrations, allowing the calculation of IC_50_ values. Scale bar = 500 µm.

Here we describe a high-content imaging dataset generated by screening a well-defined collection of 5632 compounds including 3488 with clinical or post-marketing data across 600 indications. The compounds were screened for their ability to inhibit the cytopathic effect of SARS-CoV-2 in the human epithelial colorectal adenocarcinoma cell line Caco-2 using the assay design shown in Fig. 1. The compounds we used are well annotated in terms of primary and secondary targets and we therefore hope the data and meta-data presented herein will be combined with our results and the findings of other researchers to identify additional treatment options for COVID-19, including drug combinations. One important avenue for reuse is to determine whether any of these clinical-stage compounds or related molecules could safely achieve active concentrations at the principal SARS-CoV-2 infection site, the human lung epithelium. In this manner, the combination of our *in vitro* activity data with information about tissue distribution may help to determine the most promising avenues for future COVID-19 preclinical and clinical studies.

## Methods

### Compound collection

The collection of screening compounds was assembled by an external partner (SPECS) to mirror the set established by the Broad Institute^11^. The compounds were purchased from the same set of >70 high-quality suppliers identified by the Broad Institute (Dr Joshua Bitker, personal communication) and were quality controlled by liquid chromatography/mass spectrometry (LC/MS) for purity and identity (minimum purity >90%). The compounds were stored at a concentration of 10 mM in 100% DMSO at -20 °C. A curated database is available listing the compounds, indications, known primary targets and known mechanisms of action, as well as analysis tools that can help to determine the targets and mechanisms of action if unknown^11^.

### Cell culture

Human epithelial colorectal adenocarcinoma cell line Caco-2 was obtained from the Deutsche Sammlung von Mikroorganismen und Zellkulturen (DSMZ). Cells were grown in minimal essential medium (MEM) supplemented with 10% foetal bovine serum (FBS), 100 IU/ml penicillin and 100 µg/ml streptomycin at 37 °C. All culture reagents were obtained from Sigma-Aldrich.

### Virus culture

SARS-CoV-2 was isolated from samples of travellers returning from Wuhan, China to Frankfurt, Germany^12^. SARS-CoV-2 stocks used in the experiments were passaged once on Caco-2 cells and were stored at -80 °C. Virus titres were determined as TCID_50_/ml using confluent cells in 96-well microtiter plates.

### Cytopathicity assays for primary screening and concentration-response studies

The inhibition of the SARS-CoV-2 cytopathic effect was measured using the assay workflow summarized in Fig. 1^12^. Compounds were added to confluent layers of Caco-2 cells in MEM supplemented with 1% FBS in 96-well plates. For the primary screen, the final compound concentration was 10 µM (in 0.1% DMSO) and each compound was tested in singlicate. Concentration-response profiling of primary hits was performed in triplicate with eight concentrations ranging from 20 µM to 20 nM (half-log dilution factor, 0.1% DMSO final). For initial dose response studies, compounds were tested on three different plates to minimise the influence of plate effects. Later dose response studies at lower concentrations were also done on the same plate. Following the addition of compounds, cells were immediately infected with SARS-CoV-2 at a multiplicity of infection (MOI) of 0.01. Positive controls were prepared with the virus but no compounds, and negative controls were prepared without the virus. After 48 h, the medium was removed and cells were fixed in 3% paraformaldehyde in phosphate-buffered saline (PBS). The plates were sealed and disinfected to inactivate the virus^13^ then screened by high-content imaging using the Operetta CLS platform (PerkinElmer). The image data were processed as described below and deposited on the Image Data Resource^14^.

### Image acquisition and analysis

**Fig. 2:**
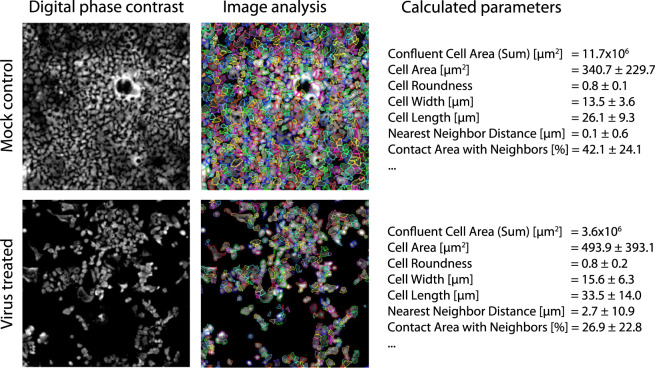
Analysis workflow. The analysis workflow is shown using mock and virus control. Images are magnified from representative fields of view. Digital phase contrast images were acquired using 10x and nine fields of view per well and subjected to image analysis using Columbus v2.9.0 (PerkinElmer). Calculated parameters offered are derived from the wells the pictures presented were taken and are given as mean with standard deviation except *Confluent Cell Area* which is given as sum of all nine fields of view per well. Additionally, the parameters refer only to cells recognized by the software. *Confluent Cell Area (Sum)* was used for the calculation of % effect, but other parameters were deposited as well and images can be downloaded and reanalysed from Image Data Resource at (https://idr.openmicroscopy.org under accession number idr0094).

Images were acquired using a 10× objective with nine imaged fields per well in label-free mode by digital phase contrast (DPC). In DPC two brightfield images are acquired at different Z positions and then an overlay image is calculated showing superior contrast and intensity properties. This method improves the signal-to-noise ratio and allows for image segmentation and single-cell analysis^15^. The images were analysed using PerkinElmer Columbus v2.9.0. The analysis sequence started with cell detection (method c, common threshold 0.05, area >100 µm^2^, splitting coefficient 6.5, individual threshold 0.05, contrast >0.05) and was followed by calculating morphology, intensity and position properties as well as cell confluence (Fig. 2). The data were deposited on the Image Data Resource^14^. Although we used cell confluence as a read out parameter additional parameters were calculated and deposited as well. These parameters can be used for further analysis to include parameters related to cellular stress or cytoskeletal impairment, both critical for viral replication. Well-level data were analysed in ActivityBase (IDBS) and R v3.6.1, using cell confluence as final read out parameter. Test results were normalized to the corresponding intra-plate virus-free negative control (assigned as 100% inhibition of cytopathicity) and virus-plus positive control (assigned as 0% inhibition of cytopathicity). Outliers were eliminated according to the three-sigma method. Plate-level statistical performance was assessed using the Z’factor calculation with values between 0.35 and 1.0 representing an acceptable assay^16^. Data for the primary screen were deposited on ChEMBL^17^.Concentration-response curves were fitted in GraphPad Prism v8.0.0 (GraphPad Software) using a four-parameter logarithmic (least squares fit). Data for the dose-response screen were deposited on ChEMBL^18^.

### Analysis of compound toxicity

Cellular toxicity was determined by measuring intracellular ATP concentrations. Caco-2 cells were harvested using Acutase followed by resuspension to the required number of 2000 cells/well in Eagle’s minimal essential medium (EMEM) supplemented with 10% FCS, 1% non-essential amino acids, 2 mM l-glutamine, 100 U/ml penicillin, 0.1 mg/ml streptomycin, and 1 mM sodium pyruvate. We transferred 20 µl of the cell suspension from each well to white solid 384-well CellStar plates (GreinerBio) and incubated the cells for 20 h at 37 °C in a 5% CO_2_ atmosphere. We then added compounds to the cells using an Echo 550 R Liquid Handler. Assay plates were incubated for an additional 48 h at 37 °C in a 5% CO_2_ atmosphere. We then prepared the CellTiter-Glo Luminescent Cell Viability Assay reagent (Promega) according to the manufacturer’s protocol. The assay plates were equilibrated at room temperature for 10 min then supplemented with 10 µl/well of the CellTiter-Glo reagent. The assay plates were centrifuged for 1 min at 700 × g and incubated for 10 min in the dark at room temperature before luminescence measurements were recorded on an EnVision plate reader (Perkin Elmer), with a measurement time of 0.05 s per sample. The image data were analysed using ActivityBase, outliers were eliminated according to the two-sigma method, and the data relating to each compound were normalized to the high (0.5% v/v DMSO, 100% viability) and low (25 µM sodium selenite, 0% viability) controls, 16 wells each, of the corresponding plates^19^. All data were generated as triplicates. The cytotoxicity data were deposited on ChEMBL^20^. The combination of cytotoxicity and cytopathic effect data allowed us to calculate the cytotoxic index (the gap between the cytopathic effect and cytotoxicity) which was also deposited on ChEMBL^21^.

### Hit selection and definition of hit

Cytopathicity assays were conducted in 96 well Cellstar plates (Greiner bio-one) using 4 wells with cells without virus (negative control) and 4 wells with cells and with virus (positive control). A three-sigma invalidation was performed on the raw data of the controls on each plate, resulting in maximal one invalidated well per plate during primary screening. During hit confirmation and dose-response studies no wells were eliminated as all wells were within three-sigma. After invalidation the plate specific z’factor was calculated and the plate was removed if quality criteria (z’factor > 0.35) were not met. Plates passing quality control were analysed and normalized as specified above. Hits were identified by having at least 75% efficiency in blocking viral cytopathicity at 10 µM. These data are available as stated in in data records section. All hit compounds were subjected to dose response studies and the 67 most active ones were followed up in dose-response studies. These data were also made available as stated in in data records section.

## Data Records

### Data structure and repositories

Five datasets were generated during this study. The first contains all the microscopy images and raw data acquired during primary screening and dose response studies in the context of the cytopathicity assay and was deposited on the Image Data Resource (https://idr.openmicroscopy.org) under 10.17867/10000148 and accession number idr0094^14^. Digital object identifier for primary screening data and the associated raw data at IDR is 10.17867/10000148a. Digital object identifier for dose response screening data and the associated raw data at IDR is 10.17867/10000148b. These datasets contain the variables listed in Table 1. The four other datasets describe calculated compound effects and were deposited on ChEMBL (https://www.ebi.ac.uk/chembl/) under accession numbers CHEMBL4303805 (cytopathicity, primary screen)^17^, CHEMBL4303806 (dose-response)^18^, CHEMBL4303807 (cytotoxicity data)^20^ and CHEMBL4303808 (cytotoxicity index – the ratio between the cytopathic effect and cytotoxicity)^21^. These accession numbers are aggregated using the Document ChEMBL ID CHEMBL4303101. The variables of these ChEMBL entries are explained in Table 2.

**Table 1:** Variables used for the dataset deposited in the Image Data Resource (https://idr.openmicroscopy.org) under accession number idr0094^14^.

Variable	Unit	Explanation
Plate	Dimensionless	Unique plate identifier.
Well	Dimensionless	Unique well identifier. For 96-well plates it consists of a letter from A to H, followed by a number from 1 to 12. For 384 well plates the letters range from A to P and the numbers from 1 to 24.
CompoundName	Dimensionless	Trivial name or abbreviation of the compound.
SMILES	Dimensionless	Canonical SMILE of the compound structure.
IUPAC_NAME	Dimensionless	IUPAC name of the compound.
pubchem_cid	Dimensionless, one entry if available	PubChem compound ID. Identifier used by PubChem (https://pubchem.ncbi.nlm.nih.gov/) to identify compound and crosslink it with data.
unichem_link	Dimensionless, one entry per compound	UniChem compound ID. Identifier used by EMBL-EBI (https://www.ebi.ac.uk/unichem/) to identify compound and crosslink it with data.
broad_ids	Dimensionless, multiple entries per compound possible if available	Broad ID or BRD ID. Identifier developed by the Broad institute and used throughout their CMap data (https://clue.io/connectopedia/what_is_a_brd_id; https://clue.io/repurposing) to identify compound and crosslink it with data.
% Inhibition (DPC)	Real number	Inhibition of cytopathicity relative to the controls of the same plate. Rounded to the second digit after comma.
Compound a hit? (over 75% activity)	Dimensionless, yes or no entry	Describes if the compound is a hit according to initial screening criteria. Only compounds which appear as a hit, meaning reducing cytopathicity by at least 75%, were selected for dose-response studies.
Phenotype Annotation Level	Dimensionless	Describes the granularity of the dataset. All data are aggregated at well level.
NumberOfCells (DPC)	Integer	Sum of the number of cells identified in DPC images per well.

These data include the images and the raw data acquired.

**Table 2:** Compound-related variables used for the document ChEMBL ID CHEMBL4303101 deposited at ChEMBL (https://www.ebi.ac.uk/chembl/) using the accession numbers CHEMBL4303805, CHEMBL4303806, CHEMBL4303807 and CHEMBL4303808 for the specific assays.

Variable	Unit	Explanation
Molecule ChEMBL ID	Dimensionless	Unique ChEMBL identifier.
Molecule Name	Dimensionless if available	Trivial name of the compound if available.
Molecule Max Phase	Integer	Describes the maximum clinical phase a compound has reached (0 to 4).
Molecular Weight	g/mol	Calculated molecular weight of the compound. Rounded to the second digit after comma.
#RO5 Violations	Dimensionless	Calculated number of violations of Lipinskies rule of five (0 to 5).
AlogP	Dimensionless	Calculated partitioning coefficient AlogP.
Compound Key	Dimensionless	Trivial name or abbreviation of the compound. One entry per compound.
Smiles	Dimensionless	Canonical SMILE of the compound structure.
Standard Type	Dimensionless	Description of the data type: can be Inhibition, Selectivity index, CC_50_ or IC_50_
Standard Relation	Dimensionless	Relation between data type and outcome, and is represented by = for all entries.
Standard Value	Real number or integer	Selectivity index is given as an integer. IC_50_, CC_50_ and effect are given as real numbers.
Standard Units	Dimensionless, nM or %	Selectivity index is dimensionless, IC_50_ and CC_50_ values are given in nM, and effect is given in %.
pChEMBL Value	Dimensionless	The pIC_50_ value of the anti-cytopathic effect.
Data Validity Comment	dimensionless	Inhibition values beyond −10% are flagged using ‘outside typical range’ and must be considered as activators.
Comment	Dimensionless	Contains IC_50_ and CC_50_ values in µM, including errors.
Uo Units	Dimensionless	Standard unit descriptor based on Unit Ontology.
Potential Duplicate	Boolean	Can be true or false.
Assay ChEMBL ID	Dimensionless	ChEMBL ID of the dataset.
Assay Description	Dimensionless	Title of the study. Identical to the title of the article.

Only compound-related data are shown because additional biological data less variable and include 16 additional variables.

### Data sets and file types

The image data are stored as 16-bit TIFF files with a resolution of 1080 × 1080. The dataset features one channel for DPC acquired at 740 nm. All image data can be downloaded (for details see: https://idr.openmicroscopy.org/about/download.html) or inspected using an online web client at https://idr.openmicroscopy.org.

The activity data are stored as CSV files in ChEMBL and can be downloaded or analysed in combination with other datasets using the variables given in Table 2 as connectors between the datasets.

## Technical Validation

Primary screening was validated by calculating Z’factor values and all 64 plates passed the quality control threshold (Z’factor > 0.35). The average Z’factor value in the primary screen was 0.72. Pharmacological validation of the assay was carried out using four compounds previously reported to inhibit the cytopathic effects of SARS-CoV-2, SARS-CoV or MERS-CoV in phenotypic readouts using the African green monkey kidney epithelial cell line Vero-E6, namely cycloheximide, emetine, remdesivir and chloroquine. Our data are compared to the results from previous reports in Table 3. The IC_50_ values for emetine (0.52 ± 0.09 µM) and cycloheximide (0.58 ± 0.02 µM) were higher than the reported activities of these compounds against MERS-CoV, SARS-CoV and SARS-CoV-2 (range 0.04–0.18 µM). The IC_50_ of the RNA polymerase inhibitor remdesivir (0.76 ± 0.18 µM) was similar to the recently reported value of 0.77 µM^22^. Chloroquine showed no activity in the Caco-2 cell assay (IC_50_ > 20 µM) thus contrasting with its reported micromolar activity in Vero-E6 cell assays. This may reflect the use of different (human vs monkey) cell lines or different viral strains, and was confirmed in the primary screen, where chloroquine and related compounds were mostly inactive.

**Table 3:** The observed IC_50_ values against SARS-CoV-2 for the four reference compounds (emetime, cycloheximide, remdesivir, and chloroquine) compared to previous reports describing tests against SARS-CoV-2, SARS-CoV, and MERS-CoV in phenotypic assays using Vero-E6 cells.

Com-pound	Results SARS-CoV-2 (Caco-2) IC_50_ µM	Toxicity Caco-2 CC_50_ µM	CI	Reports SARS-CoV-2 (Vero-E6) IC_50_ µM	Reports SARS-CoV (Vero-E6) IC_50_ µM	Reports MERS (Vero-E6) IC_50_ µM
emetine	0.52 ± 0.09	1.13 ± 0.5	2		0.051^53^	0.08^54^
cyclo-heximide	0.58 ± 0.02	>20	>30		0.043^53^	0.16^54^
remde-sivir	0.76 ± 0.18	>20	>100	0.77^22^	0.069^22^	0.074^22^
chloro-quine	>20	>20	—	1.13^22^ 46.80^55^	4.4^56^	6.275^53^

Mean IC_50_ in this study determined from triplicates at eight concentrations ± standard errors. The cytotoxicity index (CI) = IC_50_/CC_50_.

The confirmed hits included six compounds (camostat, nafamostat, lopinavir, mefloquine, papaverine and cetylpyridinium) that have already been shown to inhibit SARS-CoV-2, SARS-CoV or MERS-CoV in phenotypic assays using Vero-E6, Calu-3 (human lung) or BHK-21 (hamster kidney fibroblast) cells. Our data are compared to the results from previous reports in Table 4. Camostat and nafamostat are inhibitors of the viral entry protease TMPRSS-2. The potency of camostat against SARS-CoV-2 was ~100-fold greater in our Caco-2 cell assay than in a previous study using Vero-E6 cells^23^ but similar to the results of another study with the same virus and cell line^24^, as well as studies of MERS-CoV and SARS-CoV using Calu-3 cells^25,26^. Likewise, the potency of nafamostat against SARS-CoV-2 in our Caco-2 cell assay was >500-fold greater than in previous studies using Vero-E6 cells^27,28^ but similar to the results of a previous study of MERS-CoV cytopathicity against Calu-3 cells^26^. Papaverine was eight-fold more potent against SARS-CoV-2 (in Caco-2 cells) than against MERS-CoV (in BHK-21 cells)^29^ whereas cetylpyridinium showed similar potency in both assays^29^. These data confirm that the Caco-2 assay is pharmacologically relevant and suitable for the identification of anti-cytopathic compounds with diverse mechanisms of action. Our results using Caco-2 cells are more similar to previous studies with the human cell line Calu-3 than studies using non-human cells (Vero-E6 or BHK-21), particularly for viral entry inhibitors.

**Table 4:** The observed IC_50_ values against SARS-CoV-2 of six compounds already shown to inhibit β-coronaviruses (camostat, nafamostat, lopinavir, mefloquine, papaverine, and cetylpyridinium) compared to previous reports describing tests against SARS-CoV-2, SARS-CoV, and MERS-CoV in phenotypic assays using Vero-E6, Calu-3 or BHK-21 cells.

Compound	Results SARS-CoV-2 (Caco-2) IC_50_ (µM)	Reports SARS-CoV-2 (Vero-E6) IC_50_ (µM)	Reports SARS-CoV (Vero-E6 or Calu-3) IC_50_ (µM)	Reports MERS (Vero-6, Calu-3 or BHK21) IC_50_ (µM)
camostat	0.64 ± 0.14	>50^23^ 1^24^	~1 (Calu-3)^25^	1.0 (Calu-3)^26^
nafamostat	0.04 ± 0.02	22.5^27,28^		0.1 (Calu3)^26^
lopinavir	19.11 ± 0.47	9.12^23^	17.1 µM (Vero-E6)^57,58^	8 (Vero-E6)/not active^57,58^
mefloquine	14.15 ± 10.05	4.33^23^ 8.06^55^	7.41 µM (Vero-E6)^53^	15.5 (Vero-E6)^53^
papaverine	1.1 ± 0.39			9.45 (BHK-21)^29^
cetylpyridinium	0.62 ± 0.05			0.69 (BHK-21)^29^

Concentration-dependent anti-cytopathic effects were not observed with the reference compound chloroquine although the related antimalarial mefloquine was identified in the primary screen and was moderately active in the subsequent concentration-response screen (IC_50_ = 14.1 µM). The anti-malarial drugs tafenoquine and pyronaridine were inactive as they showed < 50% inhibition in the concentration-response screen (Hit criteria was at > 75% maximum inhibition) and, along with hydroxychloroquine, were also inactive in the primary screen. This discrepancy may reflect the greater sensitivity of Vero-E6 cells to this class of compounds, or their limited solubility.

Finally, we compared our hits with the 69 compounds in clinical or pre-clinical development recently predicted to interact with druggable targets in the SARS CoV-2/human host cell interactome screen^8^. We identified 52 compounds or close analogues (Tanimoto > 0.9) in our compound library, seven of which (14%) were classified as active in our primary screen and three of which (nafamostat, pevonedistat and camostat) displayed an IC50 value < 1 µM.

## Usage Notes

In this study, we screened a drug library of 5632 compounds for antiviral activity against SARS-CoV-2, a recently emerged virus responsible for COVID-19. A total of 117 compounds belonging to multiple drug classes showed the ability to inhibit viral cytopathicity to at least 50% at compound concentrations of up to 20 µM. Four of these hits had already been shown to inhibit SARS-CoV-2, providing validation for our choice of assay. In addition, we confirmed the antiviral activity of remdesivir (IC_50_ = 0.76 µM) during assay validation, a drug targeting the viral nsp12 RNA-dependent RNA polymerase^30^ that has been authorized for COVID-19 treatment under emergency use protocols (9). We also confirmed the antiviral activity of nafamostat (IC_50_ = 0.04 µM), which inhibits membrane fusion, and camostat (IC_50_ = 0.64 µM), which blocks viral entry by inhibiting the serine protease TMPRSS2 (11). Finally, thioguanine (IC_50_ = 1.71 µM) is reported as a competitive inhibitor of the protease PL-Pro in MERS-CoV and SARS-CoV^31^. Interestingly, the related glycoside analog thioguanosine (IC_50_ = 0.78 µM) was also active in our study. These results confirm that the assay can correctly select clinically relevant compounds with evidence for activity against viral entry and/or replication-dependent mechanisms.

Among the first group of drugs initially reported to show activity against SARS-CoV were anti-malarial compounds such as chloroquine and its close relative hydroxychloroquine^28^ although larger randomized controlled trials indicated they were largely ineffective^32^. We found that chloroquine diphosphate and other anti-malarial compounds (tafenoquine, amodiaquine, lumefantrine and primaquine) were inactive in the primary assay against Caco-2 cells whereas the chloroquine analog mefloquine showed concentration-dependent activity (IC_50_ = 14.1 µM) at the highest compound concentrations. Our hits also included the antifungal compounds posaconazole, ravuconazole, chlormidazole, and ketoconazole, all showing IC_50_ values of ~2 µM, as well as sulconazole and cloconazole, showing IC_50_ values ≥ 20 µM. Antiviral activity is rarely reported for this class of compounds, but the exceptions include posaconazole, which is active against Parechovirus A3^33^. The effective concentrations are an order of magnitude higher than those reported for antifungal activity *in vitro*. Similar membrane-interacting molecules have been reported to interfere with viral fusion to the host membrane, potentially explaining the mechanism of action for this class of compounds^34^. Interestingly, a recently preliminary analysis of historical electronic health records for SARS-CoV-2 positive patients presenting with COVID-19 symptoms identified ketoconazole as a medication associated with reduced likelihood of hospital admission^35^. In terms of possible mechanisms of action, *in silico* studies predict that posaconazole binds the SARS-CoV-2 spike protein, supporting the case for more detailed epidemiological and mechanistic analysis to determine the role of these antifungal compounds in a SARS-CoV-2 setting^36^.

The crystallization of the SARS-CoV-2 spike protein complexed to angiotensin-converting enzyme 2 (ACE2), its main receptor^37^, provides insight into the viral entry mechanism. ACE2 is expressed on the apical membrane domains of Caco-2, Calu-3 and Vero-E6 cells^38^. ACE2 expression levels can be enhanced by modulating the ACE/ACE2/AT1R/TMPRSS2 system using sartans such as candesartan and telmisartan, or ACE inhibitors such as captopril and the pro-drug enalapril. Such inhibitors could either benefit or increase the risk for COVID-19 patients because AT1R inhibitors may induce ACE2 beneficially but at the expense of facilitating viral entry^39^. Our primary screen did not identify either classical ACE inhibitors such as captopril or classical AT1R inhibitor sartans.

Several drugs showing antiviral activity in our primary screen have not been shown to influence SARS-CoV-2 cytopathicity in previous studies but have been shown to inhibit other viruses. Dapivirine (IC_50_ = 0.73 µM) is a non-nucleoside reverse transcriptase inhibitor developed for the treatment of HIV, which was recently shown to possess broad antiviral activity with micromolar IC_50_ values against influenza viruses A and B *in vitro*^40^. The orally available farnesyl transferase inhibitor lonafarnib (IC_50_ = 5.68 µM) has been considered as a treatment for renal carcinoma^41^ and showed activity against hepatitis delta virus^42^. The moderately potent pre-clinical compound NSC319276 elevates intracellular Zn^2+^ levels, thus modulating the folding of p53^43^. This compound also interferes with transition metal metabolism, inducing oxidative stress and the depletion of nucleotide reserves, suggesting a possible mechanism for inhibiting viral replication.

Papaverine (IC_50_ = 1.1. µM) is a non-narcotic alkaloid phosphodiesterase (PDE) inhibitor indicated for heart disease, impotence, and psychosis, which also inhibits multiple strains of influenza virus (*25*). Similarly, ethaverine (IC_50_ = 2.15 µM) and drotaverine (IC_50_ = 6.07 µM) inhibit PDE-4^44^. Hematoporphyrin (IC_50_ = 1.85 µM) is a photodynamic activated DNA intercalator and single strand breaker with anticancer activity^45^, which also shows antiviral effects via DNA polymerase inactivation^46^. SB-612111 (IC_50_ = 0.77 µM) has been developed as a potent and selective nociceptin opioid peptide receptor antagonist^47^ and binds the opiate and adenosine receptor proteins^47^. Overall, this diverse group of compounds shares the common feature of moderate antiviral efficacy in previous assays in addition to their primary indication. Additional studies using analog, or structurally unrelated inhibitors of the same primary targets will be useful to determine whether the SARS-CoV-2 effects we observed are dependent on the compound class or target class.

We also identified an extended group of kinase inhibitors with activity against SARS-CoV-2. There are several potential explanations for the representation of multiple inhibitors from the same class, but kinase inhibitors are usually developed as oral drugs for the treatment of cancer or inflammation. They are intrinsically optimized to enter cells and are likely to act on host target pathways to exert anti-cytopathic effects. Indeed, some inhibitors developed for cancer treatment showed lower CC_50_ values than MAPKx and JNK inhibitors, which are mainly developed as anti-inflammatory drugs. For example, LY2228820, (IC_50_ = 0.87 µM) also known as ralimetinib, is a potent and selective inhibitor of the α and β isoforms of p38 MAPK *in vitro*^48^. Other MAPK kinase inhibitors, such as bentamapimod, talmapimod and RO5126766, showed IC_50_ values of 5.9 µM, 9.6 µM and 18.2 µM, respectively, but exhibited lower cytotoxicity (CC_50_ > 20 µM). Amuvatinib was one of the most potent compounds we identified (IC_50_ = 0.02 µM). It was unsuccessful in trials for the treatment of solid tumors and small cell lung carcinoma, although it was well tolerated^49^. Amuvatinib is a promiscuous tyrosine kinase inhibitor, with activity against c-MET, c-RET and the mutant forms of c-KIT, PDGFR and FLT3. Other tyrosine kinase inhibitors we identified included sorafenib (IC_50_ = 1.55 µM), regorafenib (IC_50_ = 1.67 µM), pexidartinib (5.43 µM) and vatalanib (IC_50_ = 18.27 µM) suggesting a role for kinase signaling in the receptor mediated host response to SARS-Cov-2. As well as kinase pathway modulators, we also identified a set of additional anti-cancer compounds with well-defined anti-proliferative mechanisms of action, as might be anticipated in an anti-cytopathicity assay. These compounds included the alkaloid homo-harringtonine, as well as LDE225 and LY335979 (zosuquidar).

A comparison of our primary hits to the recently proposed list of 69 interactome-targeting compounds^8^ revealed an overlapping set of 40 identical and 12 similar compounds. Four of these compounds were confirmed in the second screen with IC_50_ values < 20 µM and all but one (loratadine, IC_50_ = 15.13 µM) were among the most promising candidates with IC_50_ values < 1 µM (nafamostat, IC_50_ = 0.04 µM; pevonedistat, IC_50_ = 0.63 µM; and camostat, IC_50_ = 0.64 µM). The corresponding biochemical targets were serine proteases (nafamostat and camostat), the histamine 1 receptor (loratadine), and NEDD-8 activating enzyme (pevonedistat). The lack of activity of the predicted compound ligands, for the majority of the originally identified pathways, may reflect low target expression levels, compound solubility, or chemical/metabolic stability.

Interestingly, cyclosporine (IC_50_ = 16.7 µM) showed moderate activity in the assay, consistent with its reported activity against SARS-CoV^50,51^. Intriguingly, cyclosporine is also proposed for the treatment of COVID-19 due to its ability to downregulate host immune responses^52^. A set of apparently nonspecific antiseptic agents was identified, which is thought to act via general membrane disruption. These compounds all showed IC_50_ values < 2 µM and included cetylpyridinium, octenidine and AC1NDSS5, a sulfonium salt.

Finally, we tested several drugs indicated for the treatment of HIV infections. Among the HIV aspartyl protease inhibitors currently undergoing clinical testing for the treatment of SARS-CoV-2 infections, only lopinavir was active at the highest concentration in the concentration-response screen whereas ritonavir and darunavir were inactive in the primary screen, suggesting this enzyme class may not play a role in viral processing. Although several of the more active compounds showed a relatively small difference between cytotoxic and antiviral IC_50_ values, with cytotoxicity indices (CI) < 3 (e.g., thioguanosine, amuvatinib, cetylpyridinium, and sorafenib), detailed pharmacokinetic and human safety data are available for these compounds. Similarly, cycloheximide (IC_50_ = 0.58 µM) showed low toxicity toward Caco-2 cells (CI ~ 10) but has significant reported side effects, including DNA damage and reproductive toxicity, and is not a clinically relevant molecule. Therefore, it will be important to use all information on the safety and toxicity of compounds to establish whether a sufficiently high concentration can be achieved safely at the site of action, presumably the lung epithelium, before proposing follow-on clinical studies.

We would like make readers aware that other parallel data sets are available from the COVID-19 portal (https://www.covid19dataportal.org/) and the NCATS databases (https://ncats.nih.gov/expertise/covid19-open-data-portal). Our data should be analysed in conjunction with these complementary data sources.

## Data Availability

Images were acquired using an Operetta CLS system combined with Harmony v4.9 followed by image analysis with Columbus v2.9.0 (all from PerkinElmer). Cytotoxicity data based on the high-content images were analysed using ActivityBase v8.0.5.4 (IDBS). Data were visualised in Prism8 for Windows 64-bit version 8.0.0 (GraphPad Software) and TIBCO Spotfire Analyst v7.11.2 (PerkinElmer). A collection of R scripts and KNIME workflows for the analysis of our datasets have been made available (https://github.com/agiani99/KNIME_Screen) with no usage restrictions.
